# The Use of High-dose Insulin Infusion and Lipid Emulsion Therapy in Concurrent Beta-blocker and Calcium Channel Blocker Overdose

**DOI:** 10.7759/cureus.3534

**Published:** 2018-11-01

**Authors:** Bilal H Lashari, Artem Minalyan, Waqas Khan, Mary Naglak, William Ward

**Affiliations:** 1 Internal Medicine, Abington Hospital - Jefferson Health, Abington, USA; 2 Pulmonology and Critical Care, Abington Hospital - Jefferson Health, Abington, USA

**Keywords:** calcium channel blockers, beta blockers, lipid emulsion therapy, high-dose insulin

## Abstract

Patients admitted with the presumed coingestion of beta-blockers (BBs) and calcium channel blockers (CCBs) should be initially managed in accordance with standardized resuscitation protocols (the airway, breathing, and circulation (ABC) approach). Additionally, more specific interventions should be promptly attempted. Intravenous glucagon and calcium salts have long been used in the treatment of BB and CCB toxicities. We present a case of a severe, concurrent BB and CCB toxicity resulting in cardiovascular collapse refractory to vasopressors. The administration of high-dose insulin (HDI) and lipid emulsion therapy (LET) resulted in a significant improvement in hemodynamics with an overall favorable outcome in the patient.

## Introduction

Beta-blockers (BBs) are among the most commonly prescribed medications. They are considered a mainstay treatment for congestive heart failure and regularly used for hypertension, ischemic heart disease, and arrhythmias. Notably, BBs are also known to be effective in many other noncardiogenic conditions, in particular, migraine, essential tremor, and esophageal varices in patients with portal hypertension. On the other hand, the use of BBs has been associated with numerous side effects: (1) related to changes in hemodynamics (bradycardia and hypotension) and (2) affecting the normal functioning of other organ systems (bronchospasm, facilitation of hypoglycemia, hyperkalemia, depression, and sexual dysfunction). The intentional or unintentional overdose of BBs manifests with multiple, mostly cardiovascular, side effects [1]. Coingestion with calcium channel blockers (CCBs) can result in deleterious hemodynamic changes, which, in many instances, are challenging to reverse [2].

## Case presentation

**Figure 1:**
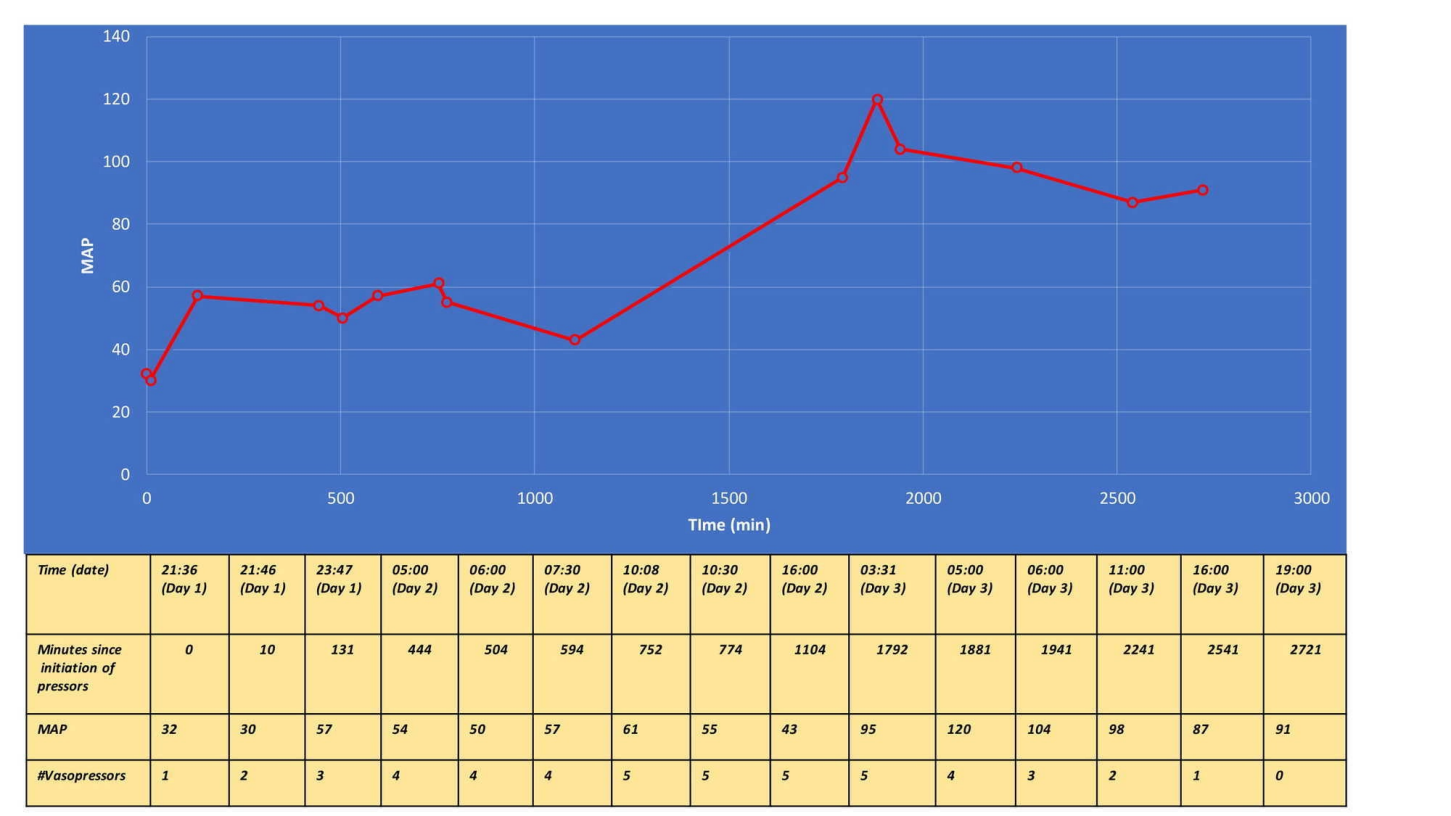
Vasopressor support and change in MAP over the course of hospitalization MAP: Mean Arterial Pressure

**Figure 2:**
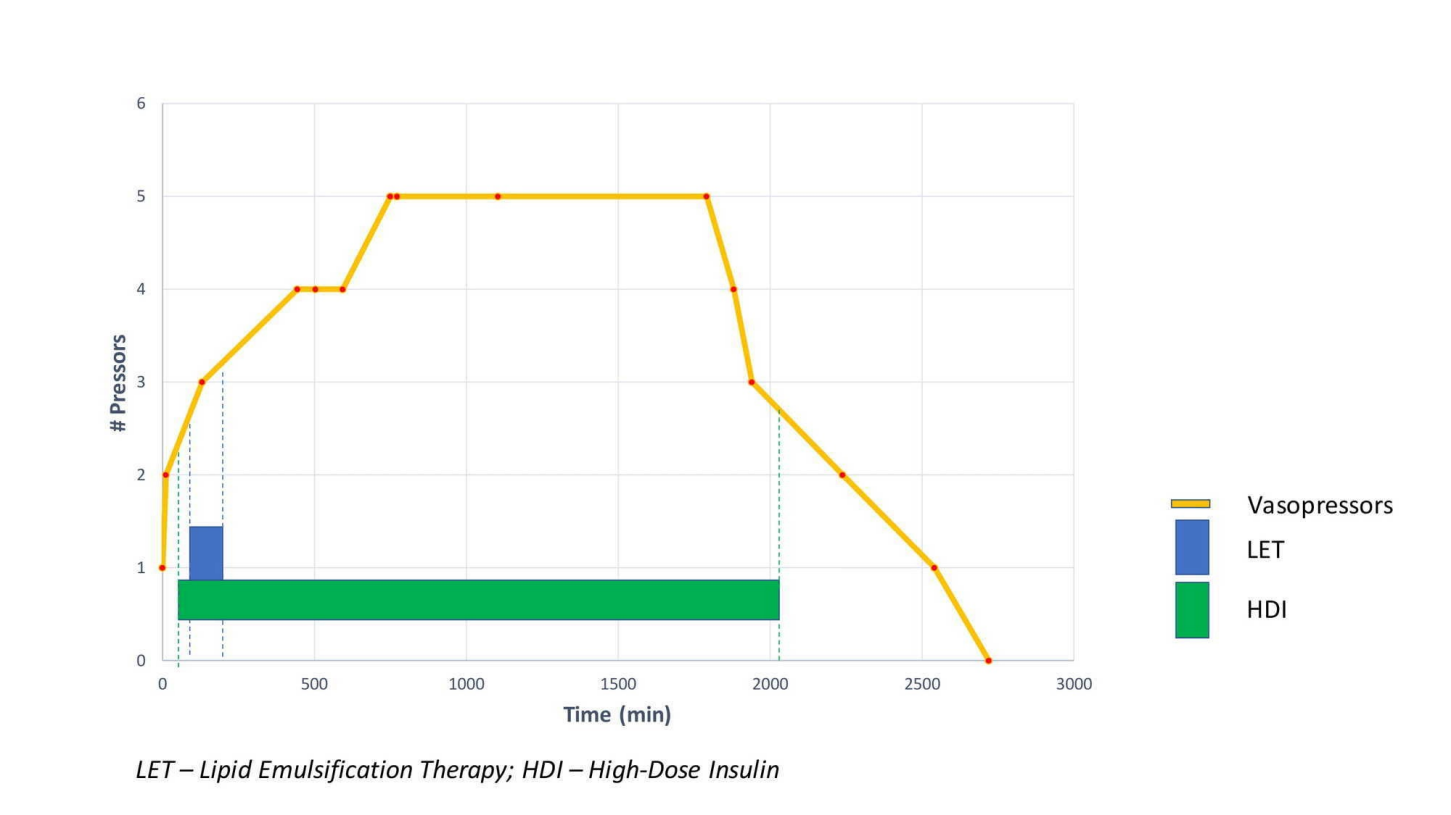
The duration of LET and HDI infusion and vasopressor support LET: Lipid Emulsion Therapy; HDI: High-dose Insulin

A 44-year-old Caucasian male with a past medical history of resistant hypertension and aortic dissection (Type B) was brought to the emergency department after he was found unresponsive in his apartment next to a suicide note. Medication history included lisinopril, clonidine, chlorthalidone, labetalol, and nifedipine. His vital signs on admission were: blood pressure (BP) 132/68 mmHg, heart rate (HR) 54 beats per minute (bpm), respiratory rate (RR) 12 breaths per minute, saturation O^2^ (SatO_2_)_ _98% on a non-rebreather mask. His level of consciousness was severely depressed, with Glasgow Coma Scale (GSC) 6. He was intubated for airway protection and given Narcan intravenously without significant changes in his vital signs or mental status. The chest X-ray was unremarkable, except for a marked widening of the mediastinum (a known history of Type B aortic dissection). A computed tomography (CT) scan of the head without contrast was also unremarkable. Repeat vital signs 30 minutes later were: BP 67/30 mmHg, HR 54 bpm, RR 16 breaths per minute, and SatO_2_ 99% on mechanical ventilation. The patient received 3 liters of normal saline intravenously along with 5 mg intravenous (IV) atropine followed by glucagon IV push and calcium gluconate IV push. After initial therapeutic interventions, his BP dropped to 45/25 mmHg and HR to 48 bpm. He was started on dopamine IV for hypotension and bradycardia and admitted to the medical intensive care unit (MICU). Central and arterial lines were placed for the administration of the vasopressors and continuous BP monitoring, respectively. Despite the initiation of dopamine infusion, the patient continued to remain hypotensive (Figure 1). Subsequently, four other vasopressors were added at their maximum infusion rates (Figure 2). In light of a suspected severe overdose with hypotension refractory to the infusion of vasopressors, the decision was made to initiate lipid emulsification therapy (LET) along with high-dose insulin infusion (HDI) (Figure 2). To prevent hypoglycemia, the patient was started on 5% and then 10% dextrose infusions. No hypoglycemia was recorded. On Day 2, the patient developed an acute kidney injury with non-anion gap metabolic acidosis. He was started on an IV bicarbonate drip. On Day 3, his hemodynamic parameters significantly improved: four out of five vasopressors were weaned off (remained only on dopamine). On the same day, IV insulin (with dextrose) and bicarb infusions were discontinued as well. On Day 4, the patient was off all vasopressors, and he was extubated on Day 5. He was transferred to inpatient psychiatric rehabilitation on Day 7.

## Discussion

Beta-blockers

Apart from beta-receptor-related toxic effects on cardiac muscle (bradycardia, decreased contractility, and atrioventricular conduction), a combination of several factors can determine the severity of the overdose: (1) intrinsic sympathomimetic activity (ISA), which is characterized by agonistic effect at lower levels and receptor antagonism at higher levels (labetalol); (2) membrane stabilizing activity by inhibiting cardiac fast sodium channels, which, in turn, leads to QRS prolongation (the most deleterious characteristic); (3) lipid solubility (the higher the solubility, the more likely a drug will cross the blood-brain barrier) [3]. Needless to say, pharmacokinetics (including half-life and site of metabolism) plays an important role in predicting the potential toxicity of an individual beta-blocker. The diagnosis of BB toxicity should be suspected in all cases of bradycardia and hypotension, which are the two most common findings on presentation. Other typical manifestations include hypoglycemia, bronchospasm, and mental status changes. The severity of the latter complication is directly proportional to the level of lipophilicity of an individual beta-blocker. “Bradyarrhythmias” is an umbrella term that best characterizes EKG findings in patients with BB toxicity. Importantly, a prolonged PR can be an early sign of BB or CCB toxicity. It becomes especially helpful when bradycardia has not developed yet.

Calcium channel blockers

CCBs are a large group of medications that exert their pharmacological actions by blocking L-type calcium channels either in the vasculature (dihydropyridines) or in the cardiac muscle (non-dihydropyridines), which can cause arterial dilation with reflex tachycardia and bradycardia with decreased contractility, respectively. At higher doses, all CCBs are responsible for the depression of cardiac function. Unlike BBs, patients with a CCB overdose can remain neurologically intact. Only at higher doses, when cerebral circulation is significantly compromised, the neurological status may worsen. Another distinctive characteristic of CCBs when compared to BBs is the tendency towards hyperglycemia [4]. This phenomenon can be explained by the physiologic role of calcium, which mediates the secretion of insulin in the pancreas.

Management of BB and CCB toxicity

The initial management of concurrent overdose with BBs and CCBs is similar to any other life-threatening condition associated with altered mental status. Stabilizing airways, breathing, and circulation are three pillars of any resuscitation regardless of the cause. Profound hypotension and bradycardia are two hallmark manifestations of BB and CCB toxicity. Therefore, IV boluses of isotonic crystalloid and atropine should always be administered prior to more sophisticated interventions. In many instances, the reversal of cardiovascular toxicity cannot be achieved without a further escalation of therapeutic interventions. IV glucagon (initially tried in reversing BB symptoms) and IV calcium salts (CCB overdose) are added to IV fluids and IV atropine [5]. Given that coingestion (either intentional or unintentional) is often present, one should not be reluctant to try both IV glucagon and IV calcium salts at the same time. After a trial of all the above-mentioned therapeutic interventions, it is recommended to give vasopressors. Interestingly, the infusion of vasopressors without other interventions (IV glucagon, atropine, and calcium salts) has been found to result in a poor outcome [6]. It has been shown that very high doses of vasopressors are required to overcome cardiovascular toxicity in patients with BB and CCB overdose. At those doses, all vasopressors can exert proarrhythmogenic effects. To date, many case reports have been published, which demonstrated the effectiveness of high-dose insulin (HDI) along with glucose and lipid emulsion therapy (LET) in reversing cardiovascular toxicity in BB and CCB overdose [7-9].

High-dose insulin

Both CCBs and BBs disrupt the secretion of insulin by the beta cells of pancreatic islets. To date, the mechanism of how high-dose insulin exerts its effects on patients with BB and CCB toxicities remains an area of uncertainty. There are at least a few suggested mechanisms that are worth mentioning: (1) positive inotropic effect with vasodilation, leading to improved coronary circulation; (2) facilitated the uptake of carbohydrates by the myocardium, which is an essential source of energy for cardiac muscle cells under stress conditions; (3) inhibition of free fatty acid metabolism; (4) acceleration of lactate oxygenation, which, in turn, reverses metabolic acidosis [10-11]. In fact, the inotropic effect achieved by insulin is not catecholamine-mediated and hemodynamic improvement is more stable than with the administration of vasopressors and fluids. One should take into consideration, however, that it may take up to 60 minutes after the initiation of high-dose insulin to notice any changes in hemodynamic parameters. As expected, the side effects associated with high-dose insulin therapy include hypoglycemia, hypokalemia, and hypomagnesemia. Different protocols and calculators are widely available to assist healthcare providers with calculating the dose of dextrose (bolus and infusion) to decrease the risk of hypoglycemia. Interestingly, a dose of insulin drip as high as 10 units/kg/hr has been successfully used with an improvement in hemodynamics [12].

Lipid emulsion therapy

The role of LET in the overdose of lipophilic local anesthetics, leading to cardiovascular collapse, has been well-established. It is believed that the administration of a high dose of lipids into the blood functions as a “lipid sink” that attracts a lipophilic substance from the plasma. Of note, this creates a concentration gradient that drives a lipophilic substance away from the heart and brain (areas of high concentration) into a “lipid sink” [13]. Other described mechanisms include: (1) increased intracellular fatty acid concentration resulting in greater ATP production; (2) increased intracardiac calcium concentration; and (3) direct positive inotropic effect. It has been suggested that similar mechanisms may contribute to the promising role of LET in reversing CCB and BB toxicities [14]. There have been several case reports that demonstrated improved hemodynamic parameters (blood pressure and heart rate) in patients with BB and CCB overdose [15]. The possible side effects associated with the administration of high-dose LET may include hypertriglyceridemia, hypersensitivity reactions, fat embolism, drug-drug interactions, pancreatitis, and hepatitis. The above-mentioned side effects have been described in patients receiving LET as a component of total parenteral nutrition.

## Conclusions

BB and CCB coingestion is a life-threatening condition that requires the prompt initiation of comprehensive resuscitation. In many instances, the escalation of “rescue” interventions is warranted, which implies the administration of HDI and LET. Implementing detailed protocols (with dosages and monitoring parameters) with assistance from a local poison control authority should be universally encouraged. To date, there are no prospective randomized trials evaluating the efficacy and safety of HDI and LET. Despite that, in our case report, we have shown that cardiovascular collapse caused by BB and CCB coingestion can be safely reversed by the administration of HDI and LET. This finding supports the data from clinical case reports published in the medical literature.
